# Uterine tumor resembling ovarian sex cord tumor with extensive rhabdoid differentiation: A case report

**DOI:** 10.1515/biol-2025-1104

**Published:** 2025-05-20

**Authors:** Rumeng Yang, Liwei Liu

**Affiliations:** Institute of Pathology, Tongji Hospital, Tongji Medical College, Huazhong University of Science and Technology, 1095 Jiefang Avenue, Wuhan, 430030, Hubei, China

**Keywords:** uterine tumor resembling ovarian sex cord tumor, striated muscle, ESR1-NCOA3, fusion gene, pathology

## Abstract

Uterine tumor resembling ovarian sex cord tumor (UTROSCT) is a rare uterine mesenchymal tumor, which is classified as a tumor with uncertain malignant potential. In this case report, we reported a 52-year-old woman who was diagnosed with UTROSCT through pathological examination of uterine intramural nodules. We reported the diagnosis and treatment process, aiming to provide new evidence and insights for the diagnosis and treatment of this rare disease.

## 1 Introduction

Uterine tumor resembling ovarian sex cord tumor (UTROSCT) was first reported by Clement and Scully in 1976, which was a rare mesenchymal tumor of the uterus, mainly affecting perimenopausal and postmenopausal women [1]. UTROSCT exhibits diverse tissue morphology, including nest-like, cord-like, sheet-like, trabecular, or reticular structure. It usually shows varying degrees of expression of epithelial, sex cord, smooth muscle, and endometrial stromal markers [1]. The tissue origin of UTROSCT remains unclear, and it typically lacks JAZF-1-SUZ12 gene fusion. A recent study has revealed that most UTROSCT cases have a unique NCOA1-3 gene rearrangement, offering a new approach for clinical diagnosis [2]. Although most UTROSCTs have a favorable prognosis, there are still a few cases of recurrence and metastasis. Therefore, UTROSCT is classified as a tumor with uncertain malignant potential [3].

UTROSCT is relatively rare, and the current number of reports is still insufficient. As a result, its diagnosis and treatment still require further investigation. This study reported a case of UTROSCT with significant rhabdoid differentiation. We discussed the clinicopathological features and differential diagnosis of this tumor in combination with the literature, with the hope of contributing new evidence and perspectives to its diagnosis and treatment.

## 2 Case report

A 52-year-old female patient underwent a B-ultrasound examination in June 2021. The results showed that nodules measuring 4.1 × 3.4 cm and 4.9 × 3.6 cm were present on the posterior and anterior walls of the uterus, respectively. The uterine cavity was centered, and a mixed echo with a visible range of 6.1 × 4.9 × 1.5 cm was detected, while the adnexa showed no abnormalities. Subsequent pathological examination suggested endometrial stromal nodules with slightly active cells, and follow-up was recommended.

In July 2021, due to “endometrial hyperplasia”, the patient underwent total hysterectomy and bilateral salpingectomy at a local hospital. During the operation, intramural nodules were observed. The section of the nodules was grayish-yellow, without necrosis. The postoperative pathology indicated epithelioid leiomyoma.

**Figure 1:**
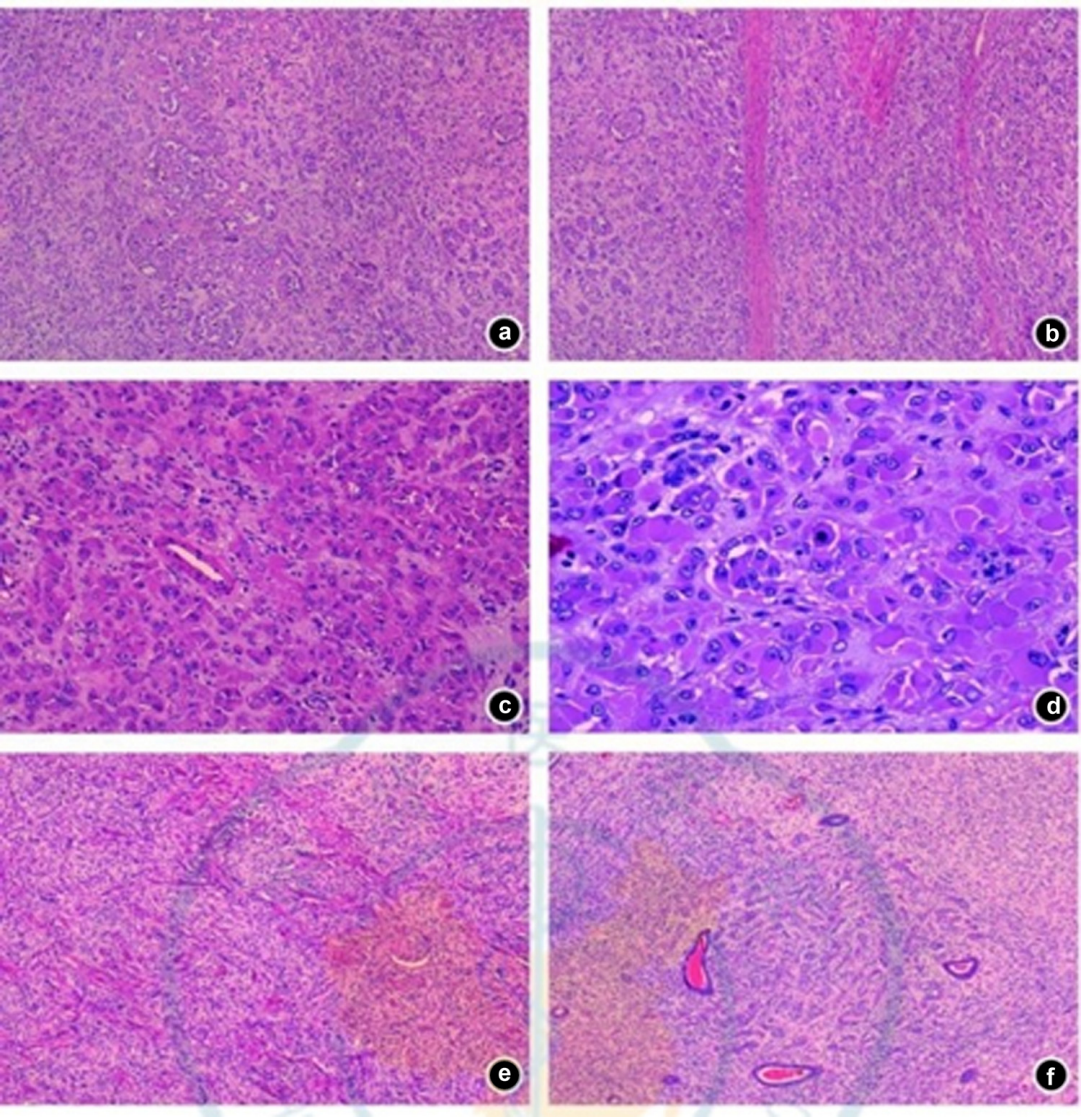
Pathological examination results (HE staining). (a) Pseudoadenoid structure; (b) normal smooth muscle interpenetration among tumor cells; (c) tumor cells showed a rhabdoid pattern, with abundant eosinophilic cytoplasm, eccentric nuclei, vacuoles, and prominent nucleoli; (d) occasional mitosis; (e) short spindle cells and vortex structure; (f) tumor tissues around the endometrial glands were arranged in a cord-like manner.

Subsequently, the patient came to our hospital for a pathological consultation. The results were as follows: (1) Most rhabdoid cells with abundant cytoplasm were diffusely distributed, and pseudoglandular structures were observed in focal areas (Figure 1a). Normal smooth muscle was interspersed among the tumor cells (Figure 1b). (2) The tumor cells had a mild appearance, abundant cytoplasm, large nucleoli (Figure 1c), and occasional mitotic figures (Figure 1d). (3) Short spindle cells formed a whorled structure (Figure 1e), and in focal areas, there was a diffuse whorled tumor (Figure 1f).

**Figure 2:**
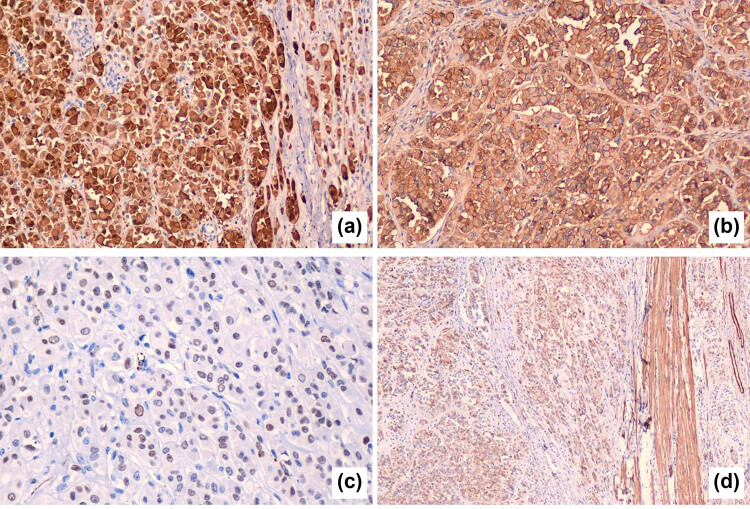
Immunohistochemical results of the tumor: (a) sex cord markers Calretinin, (b) CD56, (c) WT-1, and (d) SMA.

The immunohistochemical results were as follows: (1) Vimentin, pancytokeratin (PCK), and cytokeratin 8/18 (CK8/18) were positive, while epithelial membrane antigen (EMA) was negative. (2) Sex cord markers, including Calretinin (Figure 2a), CD56 (Figure 2b), CD99, and Wilms tumor 1 protein (WT-1) (Figure 2c), were positive. (3) Steroidogenic factor-1 (SF-1), inhibin, and Melan-A were negative. (4) Desmin (DES) and smooth muscle actin (SMA) were focally positive (Figure 2d). (5) Caldesmon, CD10, human placental lactogen (HPL), p63, p40, GATA-binding protein 3 (GATA-3), inhibin, human chorionic gonadotropin (HCG), S100 protein, human melanoma antigen (HMB45), and paired box gene 8 were negative.

Next-generation sequencing of soft tissue tumors (covering 507 genes) detected an ESR1-NCOA3 gene fusion mutation in the patient’s tumor tissue.

Combining the results of morphology, immunohistochemistry, and molecular genetic testing, the patient was finally diagnosed with UTROSCT with extensive rhabdoid differentiation. The patient’s general condition was good after the surgery, and no additional treatment was administered. As of the follow-up in October 2021, the patient had no discomfort, and no tumor recurrence or metastasis was detected.


**Informed consent:** Informed consent has been obtained from all individuals included in this study.
**Ethical approval:** The research related to human use has been complied with all the relevant national regulations, institutional policies and in accordance with the tenets of the Helsinki Declaration, and has been approved by the Ethics Review Committee of Tongji Hospital.

## 3 Discussion

UTROSCT commonly occurs in middle-aged women (with a median age of 50 years). The main clinical symptoms are abnormal vaginal bleeding or pelvic pain [1]. Most patients present with an enlarged uterus or an obvious uterine mass, although some UTROSCT cases may be incidentally discovered [1]. Grossly, most UTROSCTs appear as intramural nodules, with a few located in the submucosa and protruding into the uterine cavity. The average diameter of the tumor is approximately 6 cm, and the section is yellow or brown. UTROSCTs are mainly solid, and a few can be cystic-solid. They usually have clear boundaries, while a few have irregular edges. Microscopically, most UTROSCTs show diffuse growth, and a few can be nodular. Tumor cells are often arranged in a variety of mixed patterns, including sheet-like, cord-like, nest-like, island-like, trabecular, tubular, or reticular growth. The tumor cells have a mild morphology, often a mixture of spindle or epithelioid shapes, and exhibit diverse nuclear and cytoplasmic features. The tumor cells usually contain a small amount or abundant eosinophilic cytoplasm. Transparent, vacuolar, lipid-like, and signet ring-like changes can also be observed. In rare cases, they may show a rhabdoid pattern [2]. Mitotic figures are generally rare (<5/10 high-power fields), but there are occasional cases with a significant increase. In recurrent cases, the number of mitotic figures can be significantly elevated, but no pathological mitotic figures are observed [4]. The tumor stroma is scanty, showing hyalinization and collagenization. In some cases, it can be loose and edematous or undergo mucinous degeneration. In a few cases, obvious lymphocyte infiltration can be seen [2]. In this case, the UTROSCT mainly presented in a rare rhabdoid pattern, with spindle-shaped tumor cells and few typical sex cord-like components. This significantly increased the difficulty of diagnosis.

UTROSCT has various immunohistochemical expression patterns and usually expresses some sex cord markers. Stewart et al. [5] suggested that Calretinin is the most sensitive marker for UTROSCT, but it is often focally expressed. In contrast, SF-1 has the highest specificity. Recent studies have proposed that Calretinin, inhibin, CD99, Melan-A, and WT-1 can be used as a group of detection markers. The positive expression of Calretinin and at least one of the above markers is a necessary condition for the diagnosis of UTROSCT [6]. In this case, Calretinin, CD56, CD99, and WT-1 were mainly expressed, which is consistent with its immunohistochemical characteristics.

The extensive rhabdoid differentiation in this UTROSCT case is an extremely rare feature. In comparison with most reported UTROSCT cases, which usually present with more typical sex cord-like components and growth patterns, this case stands out. This unique feature significantly impacts the diagnosis and differential diagnosis. Morphologically, the dominance of rhabdoid cells and the scarcity of typical sex cord-like structures make it challenging to diagnose this case as a UTROSCT based on traditional morphological criteria alone. In routine practice, the presence of rhabdoid cells might lead to misdiagnosis as other tumors with rhabdoid features, such as rhabdomyosarcoma or epithelioid malignancies. Immunohistochemistry becomes crucial in this situation. Although the immunophenotype of this case is consistent with UTROSCT in terms of positive expression of sex cord markers like Calretinin, CD56, CD99, and WT-1, the presence of rhabdoid cells requires a more comprehensive immunohistochemical panel to rule out other differential diagnoses.

Recent studies have elucidated the molecular genetic characteristics of UTROSCT through next-generation sequencing. Goebel et al.’s study showed that NCOA1-3 gene rearrangement was found in 81.8% (18/22) of cases [2]. The most common was the ESR1-NCOA3 gene fusion, and a few cases showed GREB1-NCOA1, ESR1-NCOA2, and GREB1-NCOA2 gene fusions. Additionally, several studies have confirmed that UTROSCT lacks the characteristic JAZF-1-SUZ12 gene fusion [7] or PHF1 gene rearrangement [8] of endometrial stromal tumors and does not have the FOXL2 and DICER1 gene mutations unique to ovarian sex cord stromal tumors [9]. The next-generation sequencing results of this case showed an ESR1-NCOA3 gene fusion, which is consistent with its molecular genetic characteristics. In several other reports of UTROSCT with a rhabdomyoid morphology, the tumor tissues showed an ESR1-NCOA3 gene fusion [4], which is also consistent with the results of this study. This case emphasized the importance of comprehensive diagnosis for UTROSCT. When dealing with uterine mesenchymal tumors with complex tissue structures and a lack of classical morphology, relying solely on histological appearance is insufficient. Molecular genetic testing, such as detecting the ESR1-NCOA3 gene fusion in this case, is essential for accurate diagnosis. In addition, a wide range of immunohistochemical markers should be used to exclude other similar tumors.

The differential diagnosis of this case mainly involves tumors with epithelioid features, and immunohistochemistry can assist in the identification: (1) Epithelial-derived tumors: true epithelial tumors are EMA-positive, while mesenchymal tumors with an epithelioid morphology are EMA-negative. PCK and CK8/18 have no differential value. (2) Malignant melanoma is positive for S100, HMB45, and Melan-A. (3) Rhabdomyoma is positive for DES, MyoD1, and Myogenin. (4) Epithelioid leiomyoma: DES, SMA, and Caldesmon are positive, and epithelial markers such as PCK can be focally positive. (5) Trophoblastic tumors also have the characteristic of positive broad-spectrum epithelial expression but negative EMA. However, trophoblastic cell markers such as HPL, P63, GATA-3, and HCG are positive to varying degrees. (6) Epithelioid hemangiopericytoma contains blood vessels, smooth muscle, and fat in different proportions. Immunohistochemically, HMB45 and Melan-A are positive. (7) Endometrial stromal-derived tumors: low-grade endometrial sarcoma shows invasive growth. The tumor cells are generally relatively uniform oval or short spindle-shaped, similar to endometrial stromal cells, and CD10 staining is strongly positive. In addition, most low-grade endometrial stromal sarcomas have a JAZF1-SUZ12 gene fusion or PHF1 gene rearrangement.

The treatment of UTROSCT is mostly hysterectomy, with or without bilateral salpingectomy and oophorectomy. Currently, there have been reports of successful pregnancies in UTROSCT patients who underwent fertility-sparing treatment [10]. Therefore, for young patients with fertility needs, fertility-sparing treatment can be considered after a comprehensive evaluation of the tumor’s characteristics. However, due to the uncertain malignant potential of UTROSCT, close follow-up is necessary regardless of the treatment method chosen. In addition, in terms of treatment, emerging targeted therapies are being explored. Given the specific gene rearrangements in UTROSCT, drugs targeting the related signaling pathways might be developed in the future. For example, drugs targeting the ESR1-NCOA3-related pathway could potentially be used to treat UTROSCT. However, these are still in the experimental stage, and more research is needed to evaluate their efficacy and safety.

Although the vast majority of UTROSCTs have a good prognosis, there are still a few cases of recurrence and metastasis. Thus, UTROSCT is classified as a tumor with uncertain malignant potential. Moore and McCluggage [3] followed up and analyzed the prognosis of 34 patients with UTROSCT. Among them, eight patients had extrauterine metastasis and three patients died of the tumor. Analysis of clinicopathological features showed that an increased number of tumor mitotic figures (≥2/10 high-power fields) and necrosis were associated with a poor prognosis. Goebel et al. [2] collected the follow-up information of 11 patients with UTROSCT. Only one case relapsed after the initial diagnosis, and this case had a GREB1-NCOA2 gene rearrangement. Although UTROSCT is generally considered to have a relatively good prognosis, a small number of cases do experience metastasis. The most common metastatic sites include the pelvic lymph nodes, lungs, and abdominal cavity. However, the exact mechanisms of metastasis and the factors influencing it are still under investigation. Regarding prognosis, while the long-term impact of extensive rhabdoid differentiation remains unclear, some studies suggest a potential association with a different prognosis. Some reports indicate that tumors with rhabdoid features might have a more aggressive behavior. However, in this case, the patient has not shown recurrence or metastasis during the short-term follow-up. It is possible that the extensive rhabdoid differentiation in this UTROSCT might represent a distinct biological subtype. If so, it could potentially have unique responses to treatment and recurrence patterns. Future research with a larger sample size and longer follow-up periods is needed to clarify this relationship.

In summary, this study reported a rare case of UTROSCT. The tumor tissue in this case was mainly characterized by a rare rhabdoid pattern, with fewer typical sex cord-like components, which posed challenges to the diagnosis. When dealing with uterine mesenchymal tumors with complex tissue structures and a lack of classical morphology, active molecular testing is not only beneficial for a clear diagnosis but also provides a basis for further exploring the potential relationship between different tissue morphologies and molecular characteristics. Moreover, some studies have suggested that UTROSCT with a rhabdoid pattern is closely related to late recurrence in patients [4]. Therefore, long-term follow-up of such patients is essential.
